# Pseudotumor Cerebri in a Child with Idiopathic Growth Hormone Insufficiency Two Months after Initiation of Recombinant Human Growth Hormone Treatment

**DOI:** 10.1155/2016/4756894

**Published:** 2016-02-04

**Authors:** Eleni Loukianou, Anastasia Tasiopoulou, Constantinos Demosthenous, Dimitrios Brouzas

**Affiliations:** ^1^Department of Ophthalmology, Makarios Hospital, 2012 Nicosia, Cyprus; ^2^1st University Eye Clinic, 11527 Athens, Greece

## Abstract

*Purpose*. To report a rare case of pseudotumor cerebri (PTC) in a child two months after receiving treatment with recombinant human growth hormone (rhGH) and to emphasize the need of close collaboration between ophthalmologists and pediatric endocrinologists in monitoring children receiving rhGH.* Methods*. A 12-year-old boy with congenital hypothyroidism started treatment with rhGH on a dose of 1,5 mg/daily IM (4.5 IU daily). Eight weeks later, he was complaining of severe headache without any other accompanying symptoms. The child was further investigated with computed tomography scan and lumbar puncture.* Results*. Computed tomography scan showed normal ventricular size and lumbar puncture revealed an elevated opening pressure of 360 mm H_2_O. RhGH was discontinued and acetazolamide 250 mg per os twice daily was initiated. Eight weeks later, the papilledema was resolved.* Conclusions*. There appears to be a causal relationship between the initiation of treatment with rhGH and the development of PTC. All children receiving rhGH should have a complete ophthalmological examination if they report headache or visual disturbances shortly after the treatment. Discontinuation of rhGH and initiation of treatment with acetazolamide may be needed and regular follow-up examinations by an ophthalmologist should be recommended.

## 1. Introduction

Pseudotumor cerebri is a disorder defined as an elevated intracranial pressure with normal cerebrospinal fluid composition documented by lumbar puncture and normal neuroimaging with absence of deformity, displacement, or obstruction of the ventricular system documented by computed tomography and magnetic resonance imaging. It is more common in adults and the diagnostic criteria of PTC in this age group are summarized in Box 1 [1–3].

It can also occur at any age in childhood. It is reported that 60% of children developing PTC are over the age of ten years [4]. As PTC in young children is different in clinical presentation from adolescents and adults new diagnostic criteria for PTC in prepubertal children were established (Box 2) [5].

Usual presenting symptoms in children include headache, nausea, vomiting, unilateral or bilateral transient obscurations lasting seconds, pulsatile tinnitus, double vision, blurred vision, and stiff neck [4]. In adolescents the most common presenting complaint is headache, whereas in younger children and infants it is irritability rather than headache. Headache may be throbbing and wake the patient from sleep [6, 7]. In pediatric population, PTC is associated with endocrine abnormalities, medications, viral infections, nutritional etiologies, or systemic conditions as shown in Box 3 [6].

Recombinant human growth hormone was first introduced in medical practice in 1985 [8, 9]. It is produced through recombinant DNA technology and has primarily been used in the treatment of children with idiopathic growth hormone deficiency. It has also been used to treat other conditions including Turner syndrome, delayed puberty, and empty sella syndrome [8]. Many cases of PTC were reported in children treated with rhGH since 1993 [10]. The prevalence of PTC in pediatric population receiving treatment with rhGH is approximately one hundred times greater than in the normal population [11].

## 2. Case Presentation

A 12-year-old boy presented at the Paediatric Department complaining of severe headache without any other symptoms. He had a past medical history of congenital hypothyroidism and idiopathic growth hormone insufficiency. His body mass index (BMI) was 13 and he was under thyroxine treatment. He was also on the 8th week of recombinant human growth hormone (rhGH) treatment on a dose of 1.5 mg daily (or 4.5 IU daily). On the same day he was referred to the Eye Department for further assessment.

**Figure 1:**
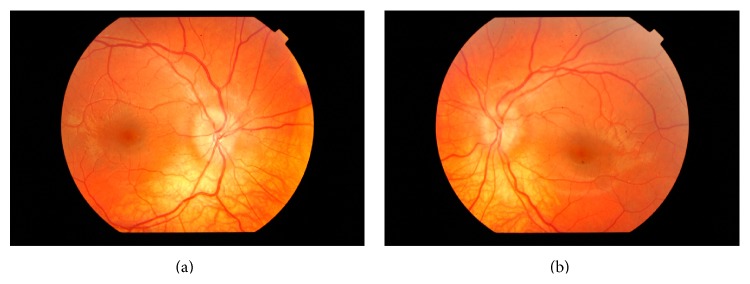
((a), (b)) Bilateral optic disc edema on initial examination. There is elevation of nasal and temporal circumference of the optic nerve with a halo surrounding the disc completely.

On initial examination his best corrected visual acuity was 6/9 in both eyes. His intraocular pressures (IOPs) were 12 mmHg in the right eye and 15 mmHh in the left. Color vision tested via Ishihara plates showed no defects in either eye. Moreover, the anterior chamber and the papillary reflexes were normal and a relative afferent papillary defect (RAPD) was not revealed in either eye. Fundoscopy revealed bilateral hyperaemic optic nerve edema, with no spontaneous venous pulsations and with increased retinal vessel tortuosity. Elevation of nasal and temporal circumference of the optic nerve obscuring some segments of the major retinal vessels and a halo surrounding the disc were apparent in both eyes (Stage 3, staging scheme of swelling of the optic nerve head by Frisen) [12] (Figures 1(a) and 1(b)).

Visual field testing was inconclusive because the patient was uncooperative. There was a second effort of visual field testing which showed an enlargement of the blind spot in both eyes without any other abnormalities. Further investigations were performed including blood pressure measurements and blood tests which were within normal limits. An assessment by a neurologist took place and a brain computed tomography (CT) scan was ordered. The CT scan showed normal ventricular size, swelling of optic nerve heads, and a partially empty sella without any evidence of mass, haemorrhage, edema, or midline shift. Lumbar puncture revealed an elevated opening pressure of 36 cm water and a normal spinal fluid composition. The diagnosis of pseudotumor cerebri was established and the young patient was admitted to the hospital. rhGH was discontinued and a treatment with acetazolamide (250 mg per os twice daily) was initiated.

**Figure 2:**
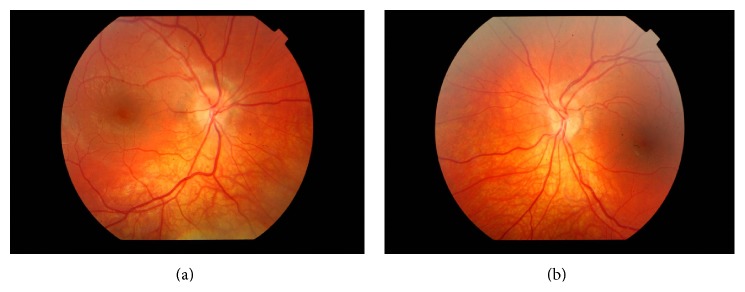
((a), (b)) Partial resolution of right and left optic nerve edema 4 weeks after discontinuation of rhGH.

The patient was reviewed regularly. Four weeks later, his best corrected visual acuity was 6/9 in both eyes. His intraocular pressures and color vision perception were normal. Fundoscopy revealed partial resolution of optic nerve edema in both eyes (Figures 2(a) and 2(b)).

**Figure 3:**
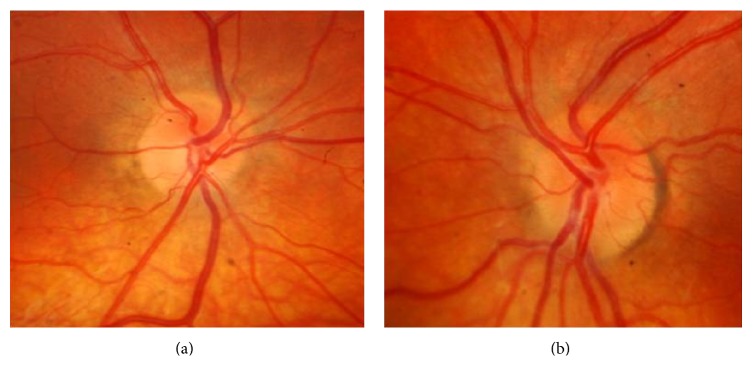
((a), (b)) Definite right and left optic nerve edema resolution after 8 weeks.

Visual field testing was repeated showing blind spot enlargement in both eyes. After discussion with the pediatric endocrinologist acetazolamide was increased to 375 mg twice daily. Eight weeks later, his best corrected visual acuity was 6/6 in both eyes. Fundoscopy showed a definite resolution of optic disc edema with spontaneous venous pulsations in both eyes (Figures 3(a) and 3(b)).

Visual field testing showed normal visual fields in both eyes. However, acetazolamide treatment (375 mg daily) was continued for four further weeks and rhGH was not started up again. The total duration of acetazolamide treatment was 16 weeks.

## 3. Discussion

The association between rhGH and the development of PTC was first reported by Otten et al. in 1992 [13]. A progressive trend in higher and more frequent doses of growth hormone resulted in an increase of reported cases [8, 14, 15]. It is well known nowadays that the rhGH-induced PTC is dose-related [16]. It is also estimated that the incidence of rhGH-induced PTC is 1, 2 per 1000 cases among the patients receiving treatment with rhGH [4]. Malozowski and Koller reported that in most cases the dosage of rhGH ranged from 0.17 to 0.35 mg/kg per week and onset of symptoms occurred from 1 week to 5 years but usually within days [1, 17–19]. In our case, symptoms occurred two months after the beginning of the treatment with rhGH on a standard dose of 1,5 mg daily. The pathogenesis of rhGH-induced PTC may involve the increased cerebrospinal fluid (CSF) production by the choroid plexus. This production is secondary to an increase in growth hormone concentrations in the CSF which induce both the local production of IGF-1 and the activation of IGF-1 receptors. Alterations in sodium and water retention mediated by acute stimulation of the renin-angiotensin system may also play an important role in the pathogenesis of the condition [1, 8, 16]. All children receiving rhGH should be monitored closely and have a complete investigation if they report headache or visual disturbances after the initiation of the treatment. Children with suspected PTC should have a careful neurologic and ophthalmologic evaluation including visual acuity assessment, color vision testing, papillary reflex evaluation, fundoscopic examination, and visual fields testing. Our patient was further investigated by an ophthalmologist and a neurologist and an rhGH-induced PTC was diagnosed. PTC usually goes into spontaneous remission. Indications for treatment are persistent headache or progressive visual deterioration due to optic nerve involvement [2]. The cornerstone of medical treatment is weight control for obese patients and cessation of any drug thought to have precipitated the condition [14]. The standard treatment includes diuretics and most notably acetazolamide [2]. In our case, the rhGH was discontinued and a treatment with acetazolamide per os was initiated shortly after a diagnosis of rhGH-induced PTC was established. In patients with severe deterioration of vision not responding adequately to medications, lumbar puncture (which could be used both diagnostically and therapeutically) and surgical procedures such as cerebrospinal fluid diversion procedure (lumboperitoneal or ventriculoperitoneal shunt) and optic nerve fenestration are recommended. In the majority of cases, resolution of both papilledema and symptoms of intracranial hypertension may take 3 to 6 months after discontinuation of rhGH. In our case, both signs and symptoms disappeared two months later. Our patient completed his treatment with decreasing doses of acetazolamide for sixteen weeks and the rhGH was not restarted. Restarting treatment on the same dose usually causes recurrence of the PTC which could be prevented if the treatment is restarted on a lower dose [8, 16]. During the follow-up period all children should be evaluated by an ophthalmologist and a pediatric endocrinologist. In our case, the close collaboration between them resulted in rapid resolution of signs and symptoms and absolute recovery of visual acuity in both eyes.

## Figures and Tables

**Box 1:**
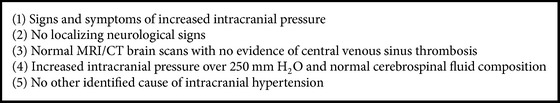
**Box 1: **Diagnostic criteria for PC in adults.

**Box 2:**
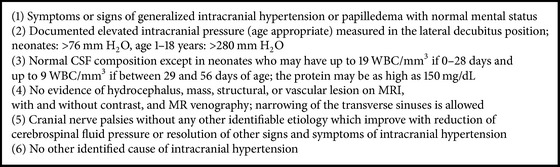
**Box 2: **Diagnostic criteria for prepubertal PTC.

**Box 3:**
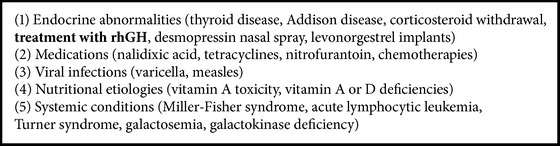
**Box 3: **Etiology of PTC in children.
